# Rescue IVM of Denuded GV- and MI-Stage Oocytes of Premenopausal Rats with Oncostatin M, Insulin-like Growth Factor I, and Growth Hormone

**DOI:** 10.3390/life12081247

**Published:** 2022-08-16

**Authors:** Yesim Akdemir, Yaprak Donmez Cakil, Belgin Selam, Mustafa Erinc Sitar, Mehmet Cincik

**Affiliations:** 1 Department of Obstetrics and Gynecology, Faculty of Medicine, Bulent Ecevit University, Zonguldak 67100, Turkey; 2 Department of Histology and Embryology, Faculty of Medicine, Maltepe University, Istanbul 34857, Turkey; 3 Department of Obstetrics and Gynecology, School of Medicine, Acibadem Mehmet Ali Aydinlar University, Unit of ART, Acibadem Altunizade Hospital, Istanbul 34752, Turkey; 4 Department of Medical Biochemistry, Faculty of Medicine, Maltepe University, Istanbul 34857, Turkey

**Keywords:** in-vitro maturation, germinal vesicle, oncostatin M, insulin-like growth factor-1, growth hormone, MII oocyte, rescue in vitro maturation

## Abstract

Immature oocytes are retrieved and matured through in vitro maturation (IVM). Maturation, fertilization rates, and embryo development via IVM are all lower than those found in vitro fertilization (IVF) cycles. We investigated the effects of oncostatin M (OSM), insulin-like growth factor-1 (IGF-I), and growth hormone (GH) in rescue IVM. A total of 111 germinal vesicle (GV) and 17 metaphase I (MI) oocytes were obtained after conventional IVF from 28 female Wistar albino rats. Denuded immature oocytes were cultured in maturation medium supplemented with OSM, IGF-1, or GH. The quantities of metaphase II (MII) oocytes matured from the GV stage were 17 of 30 (56.6%), 15 of 28 (53.5%), 10 of 30 (33.3%), and 7 of 23 (30.3%), in control, OSM, IGF-I, and GH groups, respectively. Maturation rates in control and OSM groups were higher than those in IGF-I and GH groups (*p* = 0.001). The quantities of MII oocytes matured from MI stage were 7 of 7 (100%), 4 of 4 (100%), 1 of 1 (100%), and 1 of 5 (20%) in control, OSM, IGF-I, and GH groups, respectively. Maturation rates from MI to MII stages in control, OSM, and IGF-I groups were higher than those in the GH group (*p* = 0.004). Acceptable maturation rates are observed with OSM in rat oocytes in rescue IVM.

## 1. Introduction

Oocyte maturation is a complex process, beginning with the luteinizing hormone (LH) surge and leading to the formation of a metaphase–II (M-II) arrested haploid oocyte. Controlled ovarian hyperstimulation (COH) is applied to develop numerous mature and competent oocytes in assisted reproductive technologies (ART) [1]. Nevertheless, immature germinal vesicle (GV) and metaphase I (MI) stage oocytes comprise up to 30% of retrieved oocytes [2,3,4,5,6]. The proportion of immature oocytes becomes more relevant in patients with advanced age, poor ovarian response (POR), or polycystic ovary syndrome (PCOS) by exceeding 50% of the retrieved oocytes [7]. A GV stage oocyte is characterized by a large nucleus (germinal vesicle) in prophase I of the first meiotic division and a prominent spherical nucleolus [8]. An MI oocyte forms following germinal vesicle breakdown and is identified by the absence of a visible GV or first polar body [9]. The maturity of retrieved oocytes is important for the outcome of ART, and the high incidence of immature oocytes might negatively affect clinical outcomes [2,3].

In vitro maturation (IVM) of immature oocytes is a well-known technique in ART. IVM was originally described by Edwards as the maturation of GV-stage cumulus–oocyte complexes obtained from antral follicles to MII stage in vitro [10]. Variations in IVM protocols are mainly based on the administration of human chorionic gonadotropin (hCG) or gonadotropin-releasing hormone (GnRH) agonists to improve the quantity and quality of oocytes and the success of ART [11]. So far, IVM has been widely utilized in patients with PCOS, yet there are several other reasons for its use, including POR, repeated in vitro fertilization (IVF) failure, and fertility preservation of patients with cancer [6,12].

Rescue IVM is a major IVM protocol corresponding to the IVM of immature oocytes collected from conventional IVF cycles after hCG priming [7]. The immature and “medically unusable oocytes” are cultured in complex tissue culture-like media for 4–48 h to allow spontaneous maturation of the oocytes to MII stage [13]. This spontaneous maturation is thought to cause a loss of beneficial cumulus cell metabolites, including mRNA, proteins, substrates, and nutrients, eventually resulting in a lower reproductive potential of IVM oocytes than that of IVF oocytes [14]. Therefore, rescue IVM has not been implemented in routine clinical practice. Nevertheless, several studies demonstrated reasonable clinical outcomes in hyper-responder patients [15], patients with polycystic ovarian syndrome (PCOS) [16], and patients with low functional ovarian reserve [13].

Several growth factors, such as growth hormone (GH) and insulin-like growth factor-1 (IGF-1), have been reported to favor oocyte maturation in vitro. The GH added into an IVM medium promotes the maturation of human oocytes [17,18] and those of other species such as rats [19]. Similarly, IGF-1 improves in vitro oocyte maturation in mice [20], bovine [21], canine [22], and porcine models [23]. Oncostatin M (OSM), a cytokine of the IL-6 family, is another growth factor thought to be related to oocyte maturation [24,25]. OSM and its receptors have been shown to be expressed in both oocytes and granulosa cells of ovarian follicles [26]. In the present study, we aim to investigate the possible roles of OSM, IGF-1, and GH in oocyte maturation after routine IVF retrieval following hCG administration in premenopausal rats.

## 2. Materials and Methods

### 2.1. Animals and Oocyte Recovery

The experimental protocol was approved by the Maltepe University Animal Experiments Local Ethics Committee (Protocol Number: 2019.04.01). 28 female Wistar albino rats bred at the Maltepe University Experimental Animals Laboratory (age, 14 months) were used in the study. The rats were monitored by a veterinarian throughout the experiment and were maintained at 24 ± 2 °C, 45–50% humidity and 12:12 hours light-dark cycle and were fed ad libitum.

All rats were administrated intraperitoneally with recombinant human follicle stimulating hormone (FSH; two daily injections, Gonal F, Merck, Germany) at a dose of 8 IU/kg, followed by hCG (Ovitrelle, Merck, Germany) at a dose of 100 IU/kg, 48 h later according to Lacoste et al. and Ozcan et al. [27,28]. As the rats are poor responders, the FSH dose was raised to 8 IU/kg due to our observations. Prior to our main study, several ovarian stimulation dose finding cycles were conceptualized to discover the optimum ovarian stimulation protocol. Stimulation cycles with 2, 4 and 8 IU/kg hFSH revealed that the highest follicular output was observed with 8 IU/kg.

The rats were sacrificed for collection of ovaries 18 h later to obtain preovulatory oocytes [29]. The weight of the ovaries were measured in grams. Preovulatory cumulus-oocyte complexes obtained by aspiration of follicles larger than 1 mm were transferred to LifeGlobal HTF Medium (CooperSurgical Fertility Companies, Målov Denmark). To investigate only rescue IVM approach and mimic the conventional IVF routine, we only aspirated Graafian follicles larger than 1 mm. The Graafian follicles were reported to be approximately 0.9–1.0 mm in diameter [19,30]. Hence, we excluded almost all primordial and antral follicles before denudation that would cause bias in maturity rate.

Next, the oocytes were freed from cumulus cells using the hyaluronidase enzyme (HYASE, Vitrolife—Swemed/Sweden) and a pair of 30 G insulin needles handled carefully under the microscope. Cumulus cells were cleaned with a denudation pipette.

### 2.2. In Vitro Maturation of Oocytes

**Figure 1:**
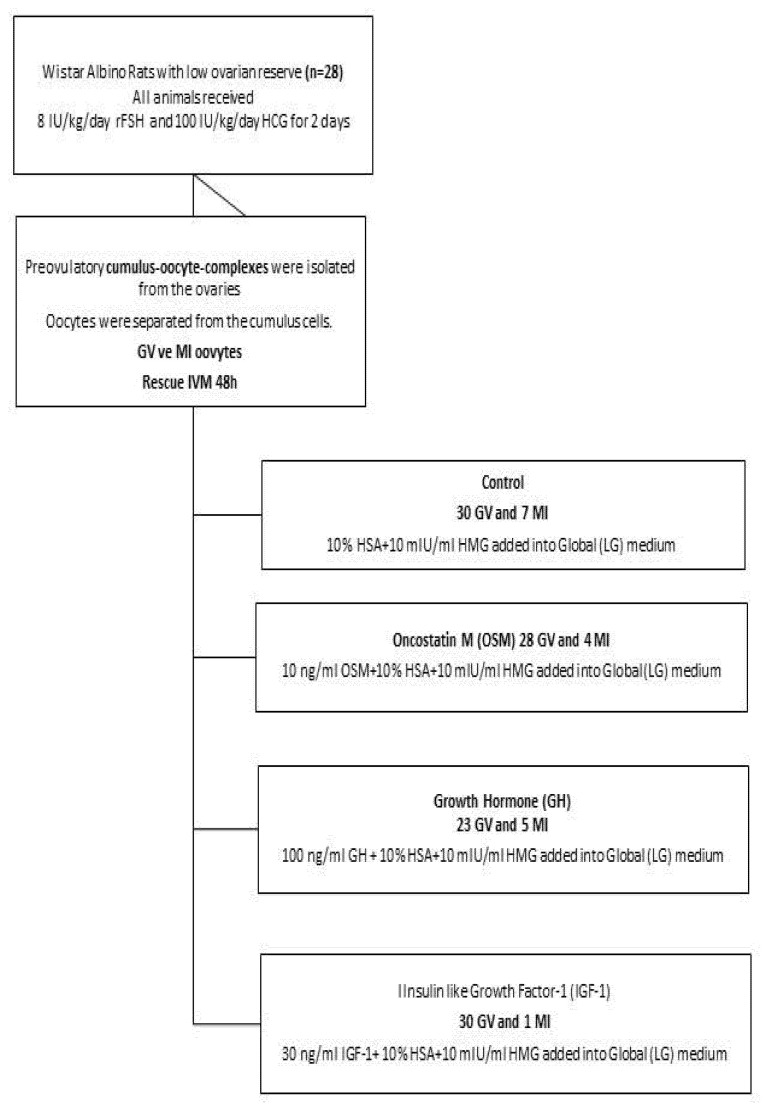
Flow chart demonstrating the experimental protocol and IVM culture conditions.

Four groups, each containing seven rats, were formed, and the GV and MI oocytes of each group were cultured in maturation medium supplemented with different growth factors (Figure 1).

The medium LifeGlobal was purchased from Cooper Surgical Fertility Solutions (USA). Six culture media droplets and one washing droplet (each 30 µL) were formed in each petri dish. The immature oocytes from each group were washed three times in phosphate-buffered saline (PBS) and they were then cultured for 48 h in: (i) control maturation medium containing 10% human serum albumin (HSA) and 10 mIU/mL menotropins (HMG, Merional, Ali Raif İlaç, Istanbul, Turkey); (ii) control maturation medium containing 10% HSA, 10 mIU/mL HMG and 10 ng/mL rat OSM (Peprotech, London, UK) [31]; (iii) control maturation medium containing 10% HSA, 10 mIU/mL HMG and 30 ng/mL rat IGF-1 (Novus Biologicals, Littleton, CO, USA) [32]; and (iv) control maturation medium containing 10% HSA, 10 mIU/mL HMG, and 100 ng/mL rat GH (Abcam, Cambridge, UK) [19,33], all cultured at 37.5 °C in a humidified atmosphere of 5% CO_2_ in air. Concentrations of OSM, IGF-1, and GH were determined according to previous studies (referenced as [19,29,31,32]). We added 10% HSA and 10 mIU/mL HMG to maturation protocol to optimize the conditions. The droplets predominantly contained 2 oocytes, except a few containing one oocyte. The droplets were covered with light paraffin oil (Ovoil 100-Vitrolife) to prevent the evaporation of the media.

### 2.3. Assessment of Oocyte Maturation

The maturation of the oocytes was evaluated under a light microscope following decellularization of the oocyte before the rescue IVM and after 48 h incubation in IVM media in the absence or presence of the growth factors.

### 2.4. Measurement of FSH and Anti-Müllerian Hormone (AMH)

Serum FSH and AMH parameters were measured using ready-for-use rat Sandwich-ELISA kits in accordance with the manufacturer’s instructions (FSH Elabscience, USA and AMH Elabscience, USA). Both the intra-coefficients of variation (CV) and the inter-CV of the analytes were measured lower than 10% in accordance with the suggestions of the manufacturer.

### 2.5. Statistical Analysis

The SPSS 19.0 was used for statistical analysis (IBM Corp., Armonk, NY, USA). The Shapiro–Wilk test was used to test normality. For a parametric approach, a one-way ANOVA test was used to compare the groups. On the other hand, for a nonparametric approach, a Kruskal–Wallis test was used to compare the groups. A Bonferroni test was performed for multiple comparison correction purposes. For all statistical comparisons, a *p* value lower than 0.05 was assumed as statistically significant.

## 3. Results

The mean values of rat weights (g), right and left ovarian weights (g), FSH (mIU/mL), and AMH (ng/mL) of the rats are depicted in Table 1 for each group. The mean values did not show any significant difference among the groups (*p* = 0.405 for mean weight, *p* = 0.580 for mean right ovarian weight, *p* = 0.237 for mean left ovarian weight, *p* = 0.103 for mean FSH, and *p* = 0.515 for mean AMH).

**Figure 2:**
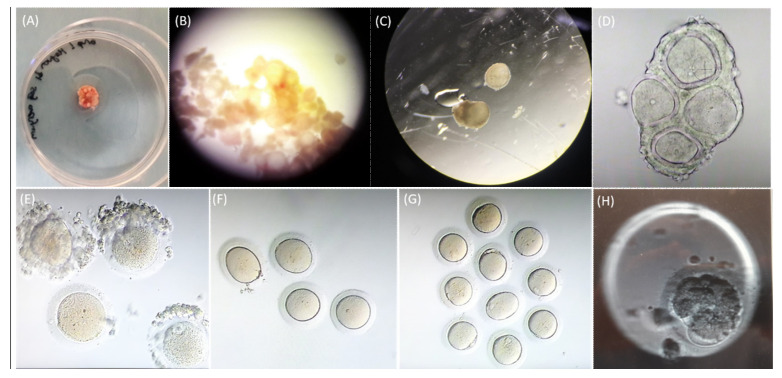
Light microscopic images of (**A**) ovarian cortex, (**B**) unruptured follicles before dissection, (**C**) unruptured follicles isolated after dissection, (**D**) oocytes following follicle rupture, (**E**) GV and MI oocytes following denudation, (**F**) MI oocytes after IVM, (**G**) MI and MII oocytes after IVM, (**H**) a parthenogenetic sample.

A total of 111 GV oocytes and 17 MI oocytes were collected from 28 female Wistar albino rats (age, 14 months) after conventional IVF stimulation and hCG administration. Figure 2 demonstrates the light microscopic images of the ovarian cortex, unruptured follicles before and after dissection, oocytes following follicle rupture, GV and MI oocytes following denudation, MI oocytes after IVM, MI and MII oocytes after IVM, and a parthenogenetic sample.

Oocyte maturation was evaluated 48 hours after in vitro culture with or without OSM, IGF-1 or GH. To determine the optimal period of IVM culture, IVM was also evaluated after 24 h of culture in the presence or absence of the growth factors (data not shown). Due to higher maturation success, 48 h was chosen as the optimal time for rescue IVM. As shown in Table 2, the percentage of the successfully rescued oocytes from GV stage to MII was similar between the control and OSM groups (56.6%-17/30 and 53.5%-15/28, respectively). On the other hand, 60% (18/30) and 69.5% (16/23) of GV oocytes remained in the GV stage when the maturation media were supplemented with IGF-1 or GH, respectively. The maturation rate from GV to MII stage was lower in IGF-1 and GH groups in comparison to control and OSM groups (*p* = 0.001).

The control and OSM groups also displayed high maturation rates from MI to MII stages (Table 3). 100% (7/7) of MI oocytes in control and 100% (4/4) of MI oocytes in OSM groups matured to the MII stage. Moreover, 100% (1/1) of the oocytes displayed maturation when the medium was supplemented with IGF-1. However, 80% (4/5) of the MI oocytes remained in MI stage when the medium was supplemented with GH. The number of oocytes that successfully reached MII stage varied significantly among the groups (0.004).

## 4. Discussion

IVM is a well-known technique in which immature oocytes are retrieved and matured in vitro. Maturation, fertilization rates, and embryo development potential of IVM are all lower than those with conventional stimulated IVF cycles [34]. Nevertheless, immature GV or MI stage oocytes could be the only ones retrieved after conventional stimulation, especially in patients with low ovarian reserve.

Rescue IVM may be a potential tool to mature immature oocytes after routine IVF retrieval following hCG administration to increase mature oocyte quantity. Although rescue IVM studies report maturation rates of 30–60% from GV to MII states, fertilization and embryo development rates of these oocytes remain unclear [35]. Few case reports reveal promising results of rescue IVM with viable embryos, but maturation rates of immature oocytes are variable. Most of the published data about rescue IVM were about retrieved immature oocytes after COH in normo-hyper-responder patients. Hatrnaz et al. [15] revealed a 65% maturation rate and 30% live births in 13 PCOS patients. The 79% maturation rate and 27% blastocyst/oocyte yield were observed in PCOS patients by Madkour et al. [16]. Similar results were detected recently by Faramarzi et al. [34]. In this study, women with endometriosis and polycystic ovaries were excluded. A total of 620 GV-state oocytes were retrieved from 310 patients after routine IVF, and 66% maturation rate, 53% fertilization rate, and 27% embryo/oocyte yield were reported. Nevertheless, a delay in early embryo development morphokinetics and an increase in embryo arrest rate were observed. Poor cytoplasmic maturation regardless of nuclear maturation state might be responsible for this negative influence. The only study [13] comparing rescue IVM results in patients with low and normal ovarian reserves revealed comparable maturation rates (72% vs. 67.8% maturation rates of GV oocytes).

IVF success is directly related to the number of mature oocytes retrieved. Therefore, every mature MII oocyte has significant value, especially in older age or POR patients and unexpectantly retrieved desynchronized oocyte cohorts. How to increase oocyte maturation rates and the number of available embryos in poor-prognosis patients is one of the main topics in IVF science.

To improve immature oocyte maturation in vitro, IVM medium is supplemented with growth factors, cytokines, or hormones [16,18,36,37]. Still, there is no consensus or routine treatment protocol about supplements in human or different species [38]. Therefore, we aimed to investigate the possible roles of OSM, IGF-1, and GH in rescue IVM of immature oocytes after routine IVF retrieval following hCG administration in low ovarian reserve premenopausal rats.

The GH plays a major role in steroidogenesis, folliculogenesis, and oocyte maturation. Its receptor has been detected in human oocytes, granulosa cells, and early embryo [39], as well as in different species including rat oocytes and granulosa cells [19,40,41]. Hassan et al. [36] found increased maturation, fertilization, and cleavage rates in human GV oocytes when cultured with GH in IVM. Menezo et al. [18] reported a case of a patient who had a healthy delivery after rescue IVM of GV oocytes with GH. However, there are conflicting results of treating denuded or cumulus-enclosed oocytes with GH in IVM for different species. Increased maturation rates of denuded mouse oocytes were observed with GH treatment in IVM [33]. On the other hand, promotion of maturation by GH was demonstrated only on cumulus-endorsed immature bovine oocytes [42,43]. Apa et al. [19] reported an increased maturation rate of cumulus-endorsed rat oocytes and no effect on denuded rat oocytes by GH. We investigated the effect of GH in rescue IVM on premenopausal denuded rat immature GV oocytes. Likewise, we observed low maturation rates (30.3% of GV oocytes were matured to MII stage) in denuded immature oocytes.

Among other growth factors that regulate ovarian functions, IGF-1 is involved in the proliferation and differentiation of granulosa cells, even in the absence of gonadotropins [20,38]. The IGF-1 induces steroidogenesis and supports early blastocyst development. Lorenzo et al. [21] demonstrated increased maturation rates of cumulus-endorsed bovine oocytes and no effect on denuded bovine oocytes with IGF-1 supplementation in IVM. Toori et al. [20] reported that IGF-1 promoted maturation rates both in cumulus-endorsed and denuded oocytes of mouse oocytes. Meanwhile, maturation rates of immature oocytes, fertilization rates, and blastocyst rates with IGF-1 were higher in cumulus-endorsed mouse oocytes than denuded oocytes. The IGF-1 receptor is present in rat oocytes and granulosa cells [41], but there are no studies evaluating the effects of IGF-1 on immature rat oocyte maturation. To our knowledge, this was the first study investigating the effect of IGF-1 on rescue IVM of denuded immature GV oocytes of premenopausal rats. We observed low maturation rates (33.3% of GV oocytes were matured to MII stage).

The IL-6-type cytokines, such as leukemia inhibitory factor (LIF) and OSM influence germ cells, follicular proliferation, and survival in human ovaries [31,44]. The LIF and OSM effects occur via GP130 shared receptors. Additionally, OSM can signal through its specific receptor, OSM receptor β (OSM-R β). The GP130 is important in meiosis and ovulation, and its expression is upregulated by initiation of meiosis in human primordial germ cells. However, OSM binds to OSM-R β with higher affinity. According to Abir et al. [26], OSM-R β was localized mostly in oocytes, and very rarely in granulosa cells in humans. OSM is suggested as a beneficial supplement to culture primordial germ cells. The OSM supplementation in IVM induced 100% cumulus oocyte complex expansion in humans, but fertilization, blastocyst, and birth rates are unknown [24]. We hypothesize that OSM might be effective in maturing immature oocytes in rescue IVM and demonstrates acceptable maturation rates (53.5% of premenopausal denuded rat immature GV oocytes were matured to MII stage). To our knowledge, this was the first study investigating the effect of OSM in maturation of denuded rat oocytes in vitro for rescue IVM.

One possible explanation for the variable maturation rates of immature oocytes in rescue IVM may be the desynchronized nuclear and cytoplasmic maturation that resulted from the early removal of cumulus cells from oocytes [7]. Denuded oocytes are confined from important substrates required for maturation. They lack the surrounding cumulus cells as the main sources of transportation via gap junctions. Retaining most of the cumulus cells with immature oocytes may also facilitate the promoting effects of supplements on maturation.

## 5. Conclusions

Our study investigated the effects of OSM, IGF-1, and GH in the maturation of denuded rat oocytes in vitro for rescue IVM for the first time. We observed acceptable maturation rates with OSM. On the contrary, in vitro maturation rates of denuded rat oocytes with IGF-I or GH were found to be lower than the rates reported in the literature. This difference could be due to aging oocytes in this study, unlike all other in vitro studies which were performed using young rats. These findings are the preliminary results of future studies that will explain mechanisms of growth factors that could be involved in the in vitro maturation process of immature denuded oocytes. Even though we present maturation rates of immature rat oocytes, the fertilization embryo development and birth rates are unknown. Interpretation of these results should be made with caution due to the probability of different effects of growth factors on different species.

## Figures and Tables

**Table 1:** Baseline demographic characteristics (mean ± SD) of the premenopausal Wistar albino rats (*n* = 28).

	Control Group	+OSM	+IGF-1	+GH	*p*
**Weight (g)**	324.28 ± 13.94	330.00 ± 3.08	344.28 ± 17.30	320.00 ± 7.86	0.405
**Right ovarian weight (g)**	106.61 ± 9.91	98.45 ± 9.09	93.47 ± 15.22	95.62 ± 9.03	0.580
**Left ovarian weight (g)**	105.82 ± 7.53	91.68 ± 9.11	78.31 ± 6.96	92.35 ± 8.19	0.237
**FSH (mIU/mL)**	7.75 ± 0.56	7.07 ± 0.25	7.81 ± 0.37	8.75 ± 0.52	0.103
**AMH (ng/mL)**	7.20 ± 0.31	7.35 ± 0.38	7.81 ± 0.43	8.06 ± 0.32	0.515

FSH = Follicle-stimulating hormone. AMH = Anti-Müllerian hormone. OSM = Oncostatin M. IGF-1 = Insulin-like growth factor-1. GH = Growth hormone.

**Table 2:** Oocyte maturation 48 hours post-incubation in maturation media supplemented with growth factors. Numbers of GV oocytes before rescue IVM and maturation rates are shown.

	No. of GV Oocytes	No. (%) of Oocytes after Rescue IVM		
		GV→GV	GV→MI	GV→MII
**Control group**	30	8 (26.6%)	5 (16.6)	17 (56.6%)
**+OSM**	28	7 (25%)	6 (21.4%)	15 (53.5%)
**+IGF-1**	30	18 (60%)	2 (6.6%)	10 (33.3%)
**+GH**	23	16 (69.5%)	0	7 (30.3%)
***p***		0.001	0.001	0.001

FSH = Follicle-stimulating hormone. AMH = Anti-Müllerian hormone. OSM = Oncostatin M. IGF-1 = Insulin-like growth factor-1. GH = Growth hormone.

**Table 3:** Oocyte maturation 48 h post-incubation in maturation media supplemented with growth factors. Numbers of MI oocytes before rescue IVM and maturation rates are shown.

	No. of MI Oocytes	No. (%) of Oocytes after Rescue IVM	
		MI→MI	MI→MII
**Control group**	7	0	7 (100%)
**+OSM**	4	0	4 (100%)
**+IGF-1**	1	0	1 (100%)
**+GH**	5	4 (80%)	1 (20%)
***p***		0.004	0.004

FSH = Follicle-stimulating hormone. AMH = Anti-Müllerian hormone. OSM = Oncostatin M. IGF-1 = Insulin-like growth factor-1. GH = Growth hormone.

## Data Availability

Data is contained within the article.
